# A sustained dual drug delivery system for proliferative vitreoretinopathy

**DOI:** 10.1080/10717544.2020.1833382

**Published:** 2020-10-26

**Authors:** Ying Xiao, Kyung Seek Choi, David Warther, Kristyn Huffman, Stephanie Landeros, William R. Freeman, Michael J. Sailor, Lingyun Cheng

**Affiliations:** a Department of Ophthalmology, Jacobs Retina Center at Shiley Eye Institute, University of California San Diego, La Jolla, CA, USA; b Department of Chemistry and Biochemistry, University of California San Diego, La Jolla, CA, USA

**Keywords:** Dual drug delivery system, proliferative vitreoretinopathy (PVR), dexamethasone, daunorubicin, rabbit retinal detachment model

## Abstract

Proliferative vitreoretinopathy (PVR) is a significant threat for vision recovery from retinal detachment or ocular trauma. Currently, no approved pharmacological intervention to prevent PVR. Daunorubicin (DNR) and dexamethasone (DEX) were sequentially loaded into oxidized porous silicon (pSiO_2_) particles by covalent conjugation. The DNR + DEX-loaded particles, and control particles loaded with DNR only and DEX only were incubated with RPE-populated collagen for daily gel surface quantitation. Toxicity was monitored by ophthalmic examinations and histological evaluation 21 days after injection. At 3rd week following intravitreal injection, a localized retinal detachment (RD) was created by subretinal injection of Healon in all pretreated eyes in addition to 3 non-interventional control eyes. 10 µg of bromodeoxyuridine (BrdU) was injected into the vitreous 4 h before sacrifice on day 3 after RD induction. Retinal sections were stained for glial fibrillary green protein (GFAP) and BrdU to identify activated glial cells and retinal cell proliferation. The studies demonstrated that all three pSiO_2_ particle types were well tolerated in vivo. DNR alone and DNR + DEX combination formulations demonstrated equally strong suppression on gel contraction (least square mean area of the gel: control = 1.71 vs. 30DNR = 1.85 or 30/40Dual = 1.83, *p* < .05). Eyes pretreated with pSiO_2_−DNR + DEX exhibited the least GFAP activation (least square mean intensity mm^−2^: Dual = 4.03, DNR = 7.76, Dex = 16.23, control = 29.11, *p* < .05) and BrdU expression (Mean number of BrdU positive cells per mm of retina: Dual = 2.77, DNR = 4.58, Dex = 4.01, control = 6.16, *p* < .05). The synergistic effect of a sustained release pSiO_2_−DNR/DEX showed promise for the prevention of PVR development while reducing the necessary therapeutic concentration of each drug.

Proliferative vitreoretinopathy (PVR) is a devastating eye disease with anomalous scarring that causes the growth of contractile membranes in the vitreous cavity, on the retinal surface, and the sub-retinal space. It is reported that PVR occurs in 5–11% of rhegmatogenous retinal detachment (RD) patients; however, it is not uncommon for PVR to come back after RD surgical interventions (Pennock et al., 2014; Kwon et al., 2016) and has a high prevalence after perforating eye globe trauma (Cardillo et al., 1997) as well as in the eyes of advanced diabetic patients (Willis et al., 2017). It is the most common cause of failure of retinal reattachment surgery and resultant blindness. In spite of advances in vitreous surgical techniques and instruments, PVR remains a challenge for medical practice.

Peri-surgical pharmacological intervention as an adjuvant therapy has been extensively investigated and holds promise to reduce PVR and enhance the surgical success rate and visual outcomes (Wubben et al., 2016). Daunorubicin (DNR) is a potent cell proliferation inhibitory agent effective against PVR (Wiedemann et al., 1998) and more potent than the commonly tested compound, 5-fluorouracil (5-FU). Although these medications are effective in experiments, the clinical application has not shown a significant reduction of PVR formation and hence there is no FDA-approved pharmacological system available for the prevention of PVR. Clinical trials for PVR prevention using DNR did not show a significant effect on the retinal re-attachment rate, though, in the DNR group, significantly fewer vitreoretinal reoperations were performed within 1 year postoperatively (Wiedemann et al., 1998). For 5-FU, two clinical trials did not show a significant preventative effect on PVR formation (Charteris et al., 2004; Wickham et al., 2007). Steroids were also investigated for the prevention of PVR and were experimentally effective. However, a recent clinical trial for PVR prevention using a dexamethasone (DEX) implant (Ozurdex) did not show a significant preventative effect (Banerjee et al., 2017).

We reasoned that there may be two aspects for the observed failure of the clinical studies. One is the lack of a controlled release system for either DNR or 5-FU. These two medications have been administered either by peri-surgical application or as a bolus intravitreal injection soon after surgery. These two drugs have short vitreous half-lives: 5 h for DNR (Kizhakkethara et al., 1995) and 150 min for 5-FU (Case et al., 1985). The other aspect may be the lack of combination therapy. PVR formation involves inflammation and aberrant cell proliferation. Although detailed interactions between these stimulating factors remain incompletely understood, ocular fluid samples from eyes with PVR clearly demonstrate high expression of inflammatory cytokines (Kaneko et al., 2018). Studies using combined therapy (anti-proliferation and anti-inflammation) in experimental PVR also suggest the potential for better outcomes (Cardillo et al., 2004; Yu et al., 2020). We have previously demonstrated that loading DNR or DEX into pSiO2 microparticles can extend their vitreous half-lives from hours to days (Hou et al., 2015; 2016). In the current study, we aimed to evaluate a platform wherein both anti-inflammatory and anti-proliferative medications were loaded into the same pSiO2 particle for sustained combination therapy. We used the DNR + DEX dual-drug loaded pSiO2 (pSiO_2_−DNR + DEX) particles on a rabbit retinal detachment PVR model and compared them to singly loaded DNR pSiO2 (pSiO_2_−DNR) and DEX pSiO2 (pSiO_2_−DEX) particles as concurrent controls to examine the possible benefit of a synergistic drug effect.

## Materials and methods

### Synthesis and drug loading of oxidized porous silicon particles (pSiO_2_)

The oxidized porous silicon particles, referred to in this work as pSiO_2_, were prepared by thermal oxidation of porous silicon particles (Hou et al., 2015, 2016). The porous silicon particles were prepared by electrochemical etching of highly doped, (100)-oriented, P++-type silicon wafers (boron-doped, 1.04 × 10^−3^ Ω cm resistivity) purchased from Virginia Semiconductors or Siltronix as we previously reported (Lewis et al., 2010). After cleaning the wafer, the porous material was obtained through electrochemical anodization in 240 mL of an electrolyte consisting of 1:1 (vol:vol) of 48% aqueous HF:ethanol (CAUTION: HF is highly toxic and can cause severe burns on contact with the skin or eyes). A temporal anodization current density profile consisting of a constant current of 30 mA cm^−2^ for 960 s, followed by a pulse of current at 176 mA cm^−2^ for 0.3 s, then 30 mA cm^−2^ for 960 s was applied. The resulting porous layer was removed from the silicon wafer substrate by replacing the electrolyte with a 1:29 (vol:vol) solution of 48% aqueous HF:ethanol and applying a current density of 6 mA cm^−2^ for 400 s. This generated a freestanding porous layer of approximate thickness 40 µm. This procedure was repeated up to 8 times per wafer. The porous Si films were then placed in ethanol and ultrasonicated for 15 min to obtain porous Si particles of approximate dimensions 40 × 40 × 20 µm. The particles were removed from the ethanol and then heated from room temperature to 800 °C in a muffle furnace (in air) at a heating ramp rate of 10 °C min^−1^. The samples were held at 800 °C for 1 h to produce oxidized porous Si (pSiO2). The pore structure of the particles was revealed by the scanning electron microscope (SEM, Phillips XL30 field emission electron microscope). The average particle size was calculated from digital photographs of evaporated pSiO2 suspension under bright field microscope. At least 200 particles from 4 random fields of view were imaged with a 5× lens and measured using ImageJ software. Pore diameter, total pore volume, and specific surface area were calculated by nitrogen adsorption/desorption experiments by the BET (Brunauer–Emmett–Teller) and BJH (Barrett–Joyner–Halenda) methods.

The subsequent functionalization of pSiO2 particles was described in detail in our previous publication (Warther et al., 2018). Functionalized pSiO2–NH–COOH particles were used for dual-drug loading. Briefly, 200 mg of pSiO_2_–NH–COOH particles were added to a solution consisting of 15.6 mL of a 136 mM aqueous solution of N-(3-dime- thylaminopropyl)-N0 -ethylcarbodiimide hydrochloride (EDC, Sigma-Aldrich) in phosphate-buffered saline (PBS) added to dimethyl sulfoxide (DMSO) in a 9:1, vol:vol ratio. This was mixed with 15.6 mL of a 13 mM solution of N-hydroxysulfosuccinimide (sulfo-NHS) in PBS:DMSO (9:1, vol:vol) and stirred for 20 min. Then, 960 μL of a 10 mg mL^−1^ solution of daunorubicin hydrochloride (Tocris Biosciences, Bristol, UK) in deionized (DI) water was added and stirred in order to create the pSiO_2_−DNR formulation. After 2 h, the particles were carefully washed with absolute ethanol and then dried under vacuum. Subsequently, 121 mg of pSiO_2_−DNR were suspended in 15 mL of CH2Cl2. 180 mg of dicyclohexylcarbodiimide (DCC) and 40 mg of 4-N,N-dimethy-lamminopyridine (DMAP) were added and rotated for 20 min. 70 mg of DEX was added and stirred for 7 days in order to ensure full coupling of DEX to the remaining carboxylic groups on the surface of the pSiO2, to form pSiO_2_−DNR + DEX. Finally, the particles were washed and dried. For the control particles, pSiO_2_−DNR (Chhablani et al., 2013) and pSiO_2_−DEX (Wang et al., 2014) were synthesized as previously reported. Particle functionalization and successful drug attachment were confirmed by Fourier Transform Infrared (ATR-FTIR) spectroscopy (Nicolet 6700 FTIR with a Smart iTR diamond ATR fixture; Thermo Fisher Scientific, Carlsbad, CA). Drug loading efficiency was quantified by thermogravimetry (TGA) (Q600 simultaneous TGA/DSC apparatus, TA Instruments, Newcastle, DL).

### *In vitro* collagen gel contraction study

Retinal pigment epithelium (RPE) is one of the major participating cells in PVR formation (Chiba, 2014). Therefore, an RPE-mediated collagen gel contraction assay was used to test cell proliferation and inhibitory effects of the test compounds (either as a single drug or in a dual-drug combination). For these studies, free drugs were used (not loaded into microparticles). Rabbit RPE cells (harvested previously in our lab) were cultured in HEPES-buffered Dulbecco’s Modified Eagle’s medium (DMEM) and Ham’s F-12 medium (1:1), supplemented with 15% fetal bovine serum (FBS), containing penicillin 100 U mL^−1^, and streptomycin 100 mg mL^−1^ at 37 °C in the presence of 5% CO2. The culture medium was replaced every 3 to 4 days. Cells were used for the gel contraction study at passage 6. 24-well culture plates were coated with 1 mL of 1% BSA/PBS for 1 h at 37 °C. Rabbit RPE cells were harvested and resuspended in complete DMEM/F12 medium. Cell-populated collagen gels (2 mg mL^−1^) were prepared by combining cold collagen type 1 (Corning, NY), 1 M NaOH, and rabbit RPE cells at a final concentration of 5 × 10^4^ cells mL^−1^ in complete DMEM/F12 media. A titration study was done for each new bottle of collagen to determine the best volume of 1 M NaOH to make a firm gel with a neutral pH as indicated by the light pink gel color. 0.5 mL of the collagen/cell solution was added to a BSA-coated well and incubated for 20 min at 37 °C under 5% CO2 to solidify the gels. 0.5 mL complete media containing either DNR, DEX, or both drugs at pre-set concentrations was gently added on top of the collagen matrix and the gels were freed by running a pipet tip around the walls of each well. Each drug concentration was tested in triplicate and two independent experiments were performed: 10 ng mL^−1^ DNR(10DNR), 30 ng mL^−1^ DNR (30DNR), 40 ng mL^−1^ DEX (40DEX), 80 ng mL^−1^ DEX (80DEX), 10 ng mL^−1^ DNR and 80 ng mL^−1^ DEX (10/80Dual), 30 ng mL^−1^ DNR and 40 ng mL^−1^ DEX (30/40Dual), 30 ng mL^−1^ DNR and 80 ng mL^−1^ DEX (30/80 Dual). The gels were incubated at 37° C under 5% CO2. The overlaying media was replaced with fresh media/drug solutions every day. Collagen gels without RPE cells were used as negative control while gels with RPE but without testing drugs served as a positive control (POS control).

The collagen gels were photographed daily with a digital camera (EOS T2i, Canon) for 10 days. The gel surface area was quantitated with NIH ImageJ 1.51 software after calibration with the 15.5 mm diameter of each well.

### *In vivo* efficacy study using a rabbit eye model of early retinal cell proliferation following localized retinal detachment

Based on our previous studies, either DNR or DEX loaded pSiO2 demonstrated good ocular safety following a 3 mg intravitreal injection. In the current study, we do not expect toxicity from the dual drug-loaded pSiO2 particles if dosing is below 3 mg because the loading rate of either drug in dual-loaded pSiO2 is lower than that of a single drug loading (Chhablani et al., 2013; Wang et al., 2014; Warther et al., 2018). For the current study, a targeted dose of 2 mg in 100 µL BSS was intravitreally injected.

Clinically encountered PVR is often late or end-stage, which no pharmacological intervention can reverse and surgical removal of the scar tissues is necessary. For a pharmacological intervention or prevention, it is important to determine cell activity toward proliferation in the early stages. Prior work has identified the upregulation of certain proteins (such as glial fibrillary green protein, GFAP, and Vimentin) in retinal cells as early as a few hours after retinal detachment (Lewis et al., 2010; Zahn et al., 2010). The current study adopted a pretreatment strategy as we described previously to test the prophylactic efficacy of the drug and delivery systems (Cheng et al., 2010).

Twelve pigmented New Zealand rabbits were divided into 4 groups and each formulation (pSiO_2_−DNR/DEX = Dual group, pSiO_2_−DNR = DNR group, and pSiO_2_−DEX = DEX group) was tested in 3 rabbit eyes while the contralateral eyes were injected with 100 µL BSS and used as controls. In addition, three rabbits were used as non-interventional controls. All animal studies were performed in accordance with the ARVO Statement for the Use of Animals in Ophthalmic and Vision Res. and were approved by the Institutional Animal Care and use Committee of the University of California, San Diego. Intravitreal injection was performed through the pars plana (2 mm from the limbus) using a 25-gauge needle and 1 mL slip tip BD syringe under anesthesia as reported previously (Chhablani et al., 2013). After injection, all remaining particles were rinsed from each vial using a syringe/needle with deionized water, dried under vacuum, and weighed for mass balance purposes. The injected eyes were examined at days 3, 7, 14, and 21 by slit-lamp biomicroscope, indirect ophthalmoscopy, and handheld tonometer (Tonopen; Medtronic, Jacksonville, FL, USA) for Intraocular pressure (IOP). Color fundus photos were taken (Canon EOS Rebel T2i camera) at each time point. Dark-adapted electroretinograms (ERG) were performed on day 21 prior to the creation of the localized retinal detachment. This study design was thus a pretreatment model, to examine if the tested formulations possessed any preventative effects. A retinal detachment was created within the test eye only (same eye as particle injection). The RD induction technique followed the procedure of Lewis et al (Lewis et al., 2009). Briefly, a subcutaneous injection of ketamine (25 mg kg^−1^) and xylazine (4 mg kg^−1^) was used for anesthesia. Eye drops of 0.25% Proparacaine (Alcon, Fort Worth, TX) were provided as additional topical anesthesia. The pupils were dilated with drops of 2.5% phenylephrine (Akorn, Lake Forest, IL) and 1% tropicamide (Akorn, Lake Forest, IL). A 20-gauge MVR blade was used to create a supranasal sclerotomy 1.5 mm posterior to the limbus. The MVR was inserted into the vitreous cavity and used to create a tiny retinotomy at the upper edge of the nasal medullary ray, 1.5 disk diameters (DD) away from the optic nerve head under the direct view of a surgical microscope. Sixty microliters of 0.25% sodium hyaluronate (Healon; Abbott Medical Optics, Santa Ana, CA) in BSS (Alcon, Fort Worth, TX) was infused through the retinotomy into the subretinal space *via* a Glaser cannula (20-gauge shaft with a 32-gauge angled tip, Eagle Labs, CA). The injection volume was determined by the formula: *V* = π/6 × *h* × (3*r*^2^ + *h*^2^), where *V* = volume to be injected, *h* = height of the detached dome of the retina, *r* = radius of the base of dome detachment. Highly elevated detachments with a narrow base will stretch the detached retina and may cause mechanical damage to retinal cells. Therefore, we used a low height and broad-based detachment. To achieve this, the injection was slow and well-controlled, allowing 1 min for the detachment to expand. Fifty microliters of aqueous humor were removed by paracentesis with a 30 G needle on a 0.3 cc BD insulin syringe prior to the induction of retinal detachment. The sclerotomy was closed by an absorbable 7-0 suture. This procedure resulted in RD of 5 ∼ 6 optic disk size created in the nasal retina, while the temporal retina was largely attached.

Color and infrared fundus photos were obtained every day to document the status of the retinal detachment, in addition to optical coherence tomography (OCT) by SPECTRALIS^®^ high-resolution, spectral-domain HRA + OCT imaging system (Heidelberg Engineering, Inc., Vista, CA; Software version: Heidelberg Eye Explorer 1.9.10.0). Three days after the creation of the retinal detachment, 10 µg bromodeoxyuridine, BrdU (BD Biosciences, San Jose, CA) in 50 µL BSS was injected into both eyes of each rabbit 4 h prior to euthanasia. Both eyes were enucleated and fixed in Davidson’s fixative for 24 h then transferred to 70% ethanol. Eyes were then grossed vertically from the middle of the optic nerve head through 6 and 12 o’clock. All the eyeballs were oriented to enable sectioning along a sagittal axis using the optic nerve as a reference.

### Histology and immunohistochemistry

The eyes were embedded in paraffin blocks and the optic nerve was used as a reference point for sectioning. Every 500 µm from the optic nerve, 15 of 5 µm sections were collected 8 times to cover 4 mm nasally from the optic nerve head. For each eye, the highest point (middle) of the dome detachment was determined from HE staining of 6 sections evenly spaced from each step.

Subsequently, two sections in the middle of the detachment and two sections 1 mm to each side of the middle were used for the immunohistochemistry staining of GFAP and BrdU.

Paraffin sections were first subjected to antigen retrieval by using Tris-EDTA buffer (PH 9.0) and heated to 95 °C for 20 min. After cooling and rinsing in PBS, sections were pretreated in blocking buffer (1:20 goat serum in PBS, 0.5% bovine serum albumin, and 0.1% Triton-X 100) at room temperature for 2 h. Subsequently, the primary antibodies were added and incubated overnight at 4 °C. Anti-GFAP primary antibody (1:400, Mouse anti-GFAP, MAB360, EMB Millipore) was used to visualize intermediate filaments within glial cells in the retina; Anti-BrdU (1:200, Rat anti-BrdU, ab6326, Abcam) was used to detect dividing cells. After three 5-min washes in PBS containing Tween-20 (PBST), the secondary antibody (1:200, Goat anti-Mouse IgG1-AF488, A21121, Invitrogen; 1:200, Goat anti-Rat IgG2a-AF546, A21133, Invitrogen) was added and incubated for 2 h at room temperature. After three subsequent washes with PBST, the slides were dried then coverslipped with Prolong Diamond Antifade mountant with DAPI (Life Technologies, Carlsbad, CA).

### Image acquisition and analysis

HE slides were viewed and imaged with an OLYMPUS BH-2 light microscope (Japan) coupled to a digital camera (Canon EOS 6 D, Japan). Immunohistochemically labeled slides were viewed and photographed using an EVOS^®^ Auto Fluorescence Imaging System (EVOS FL auto, Thermo Fisher Scientific Inc.).

For GFAP stained sections, 10× retina images were acquired from all detached retinas. For comparison, two images were taken from the non-detached retina at the equator and near the ora serrata. All images were acquired under the same conditions of brightness, contrast, and exposure time. GFAP fluorescein intensity from the retinal nerve fiber layer to the outer limiting membrane was quantitated using the ‘Auto Local Threshold – Phansalkar’ plug-in from ImageJ (Version 1.51m9). The medullary ray and inner/outer segment of photoreceptors were excluded to ensure the measurements were not influenced by autofluorescence. Mean fluorescence intensity of GFAP from each frame was expressed as pixels per mm^2^ of retina. In order to correct for background staining from different animals, the mean intensity of GFAP from the study eye was normalized by the corresponding locations of the contralateral eye. The normalized GFAP intensity from each location of each section was averaged for that eye and compared among the groups.

For BrdU quantification, 20× images were obtained from the whole section. Only when pink labeled nuclei overlapped with blue DAPI staining were cells regarded as BrdU-positive. BrdU-labeled cells were counted from the whole section, including detached area and non-detached areas, and ImageJ was used to measure the length of the retina instead of the area of the retina because some of the BrdU positive cells were found within the subretinal space, presumably due to the retinal detachment procedure. Corresponding locations from the contralateral eye were used to normalize the cell count and mean normalized cells per mm of the retina were compared among the groups.

### Statistical analysis

Continuous experimental data, IOP, ERG parameters, staining intensity of GFAP, or numbers of BrdU-positive cells per mm of the retina were expressed as means with standard deviations. For the purpose of statistical comparison between or among the study groups, the data were examined for distribution and transformed into normal distribution as needed. If the covariates were present, the least-square means of the major factors were compared while covariates adjusted. The statistical analysis was performed with JMP statistical software (JMP^®^, Version 13, SAS Institute Inc., Cary, NC).

## Results

### Characteristics of pSiO_2_ microparticles and drug loading parameters

**Figure 1.**
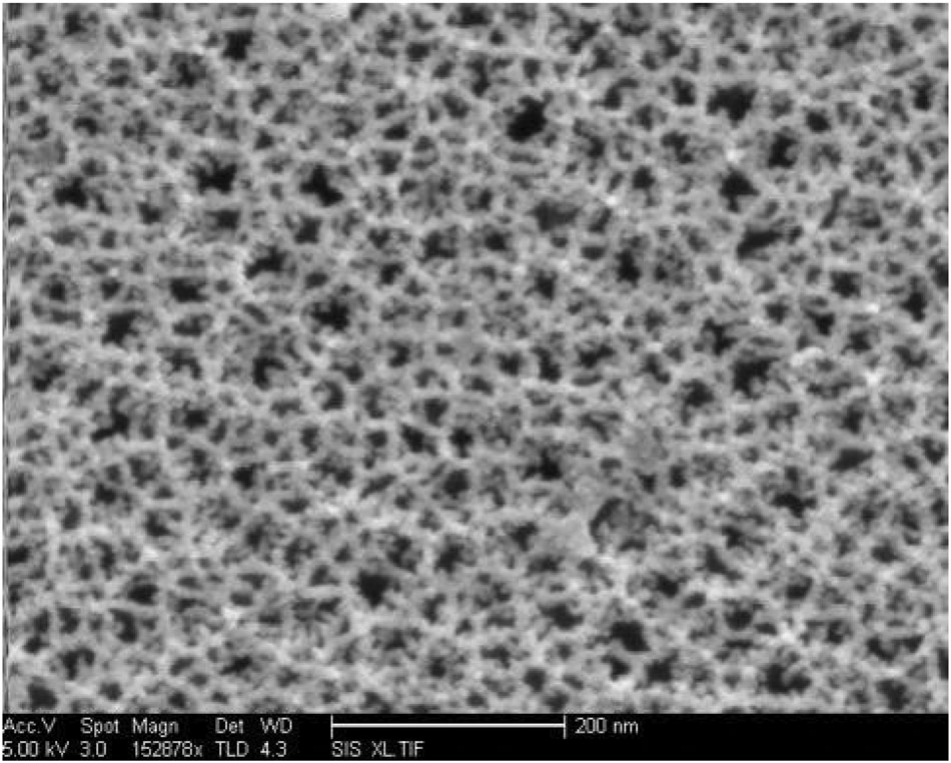
Plan-view scanning electron microscope image of a porous Si film, revealing the pore morphology. This image is of the porous Si layer. To prepare the particles used in this study, the porous Si layer was removed from the silicon wafer substrate, fractured into microparticles, and oxidized to convert the Si matrix to SiO_2_.

The average pSiO2 dimension was 40 × 40 × 20 µm. The average pore size was 26 nm and the total pore volume was 1.05 cm^3^ g^−1^, as measured by the BJH method from nitrogen porosimetry data. The surface area was calculated by the BET method as 194 ± 1 m^2^ g^−1^. Electron microscope images showed the mesoporous nature of pSiO2 microparticles (Figure 1). The nitrogen adsorption–desorption isotherm confirmed the porous nanostructure on SEM by displaying a type IV curve.

Drug loading was confirmed by FTIR and the loading rate was determined by TGA as we reported previously (Warther et al., 2018). The mass loading of DNR was 4.75% (47.5 mg g^−1^ (defined as mg drug per g particle + drug) and the mass loading of DEX was 5.91% (59.1 mg g^−1^). For the dual drug-loaded pSiO2 formulation, the DEX loading level was 2% (20 mg g^−1^) and the DNR loading level was 4.75% (4.75 mg g^−1^), making the total mass loading (of both drugs taken together) to be 6.75% (67.5 mg g^−1^).

### Collagen gel contraction on different drug/concentration combinations

**Figure 2.**
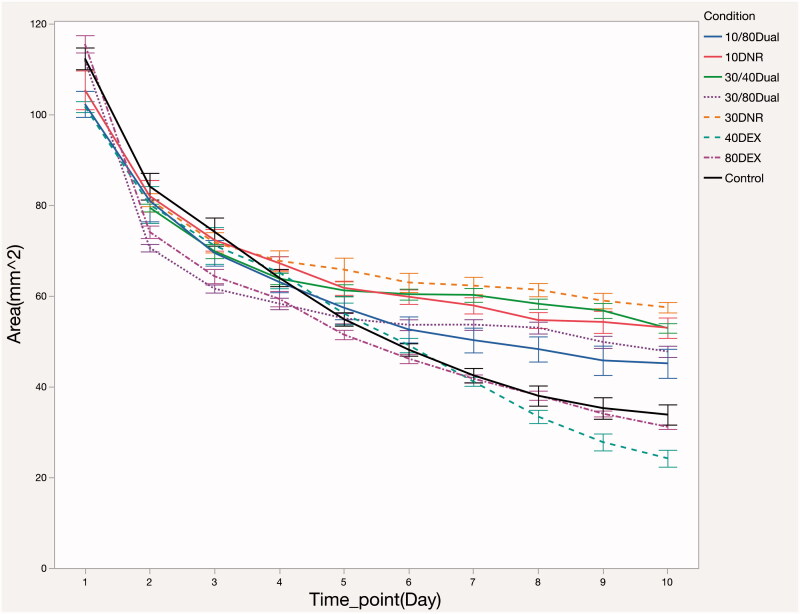
Evaluation of gel contraction mediated by embedded rabbit pigment epithelium cells as a function of time after introduction of the indicated free drugs and concentrations: 10/80Dual = 10 ng of DNR and 80 ng of DEX; 10DNR = 10 ng of DNR; 30/40Dual = 30 ng mL^−1^ of DRN and 40 ng mL^−1^ of DEX; 30/80Dual = 30 ng mL^−1^ of DRN and 80 ng mL^−1^ of DEX; 30DNR = 30 ng mL^−1^ of DNR; 40DEX = 40 ng mL^−1^ of DEX; 80DEX = 80 ng mL^−1^ of DEX.

In order to evaluate the potential synergistic effect of the antiproliferative (DNR) and the anti-inflammatory (DEX) drugs to be used in this study, the free drugs were assayed in RPE-populated collagen gels. The control gels in this assay displayed a rapid contraction phase that appeared within the first 3 days of the study, followed by a less pronounced degree of contraction for the subsequent 7 days (Figure 2).

**Figure 3.**
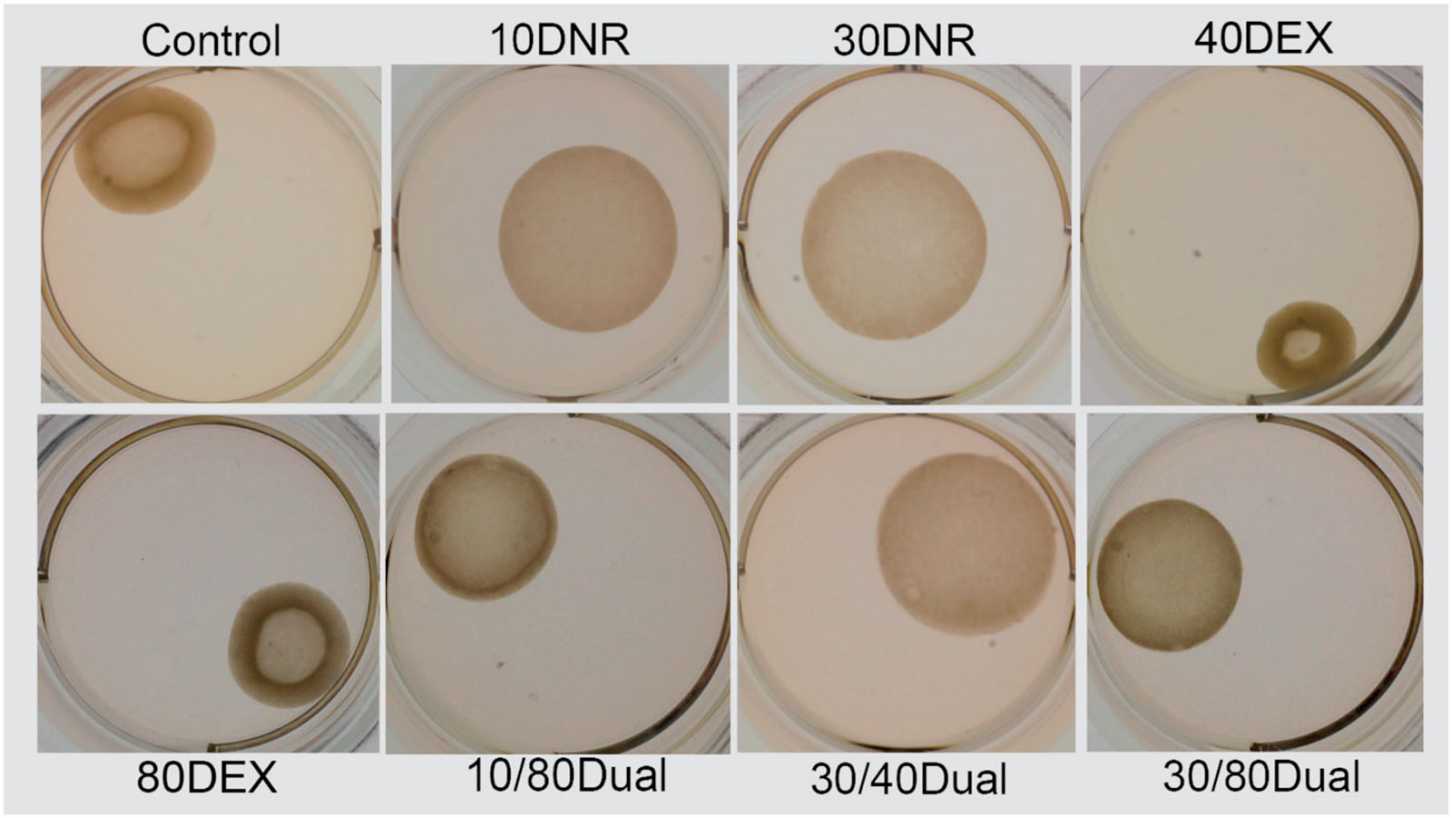
Representative images of contracted collagen gels under different treatment conditions on the 10th day.

The gel area recorded at each time point was log-transformed for normal distribution by testing conditions and the log-transformed area was regressed on testing conditions while adjusting for time points and batch of experiments. The analysis revealed that the gels incubated with 30 ng mL^−1^ DNR or 30 ng mL^−1^ DNR + 40 ng mL^−1^ DEX dual drug showed the least contraction, which was significantly different from the control and DEX-only conditions (Table 1). Representative images from each condition are shown in Figure 3. Acellular collagen gels (not shown) displayed no detectable contraction under the conditions of the study.

**Table 1.** Comparison of DNR and DEX combinations on gel contraction by least square mean differences Student’s *t*-test.

Condition	Significance			Least square Mean
30DNR	A			1.85
30/40Dual	A			1.83
10DNR	B			1.79
30/80Dual	B			1.78
10/80Dual	C			1.74
80DEX	C	D		1.72
Control		D	E	1.71
40DEX			E	1.70

Conditions as defined in Figure 2. Levels annotated with the same letter are not statistically different. Those not given the same letter are significantly different from the other groups (*⍺* = .05, *t* = 1.96).

### Clinical monitoring of the eyes injected with different drug and delivery systems

**Figure 4.**
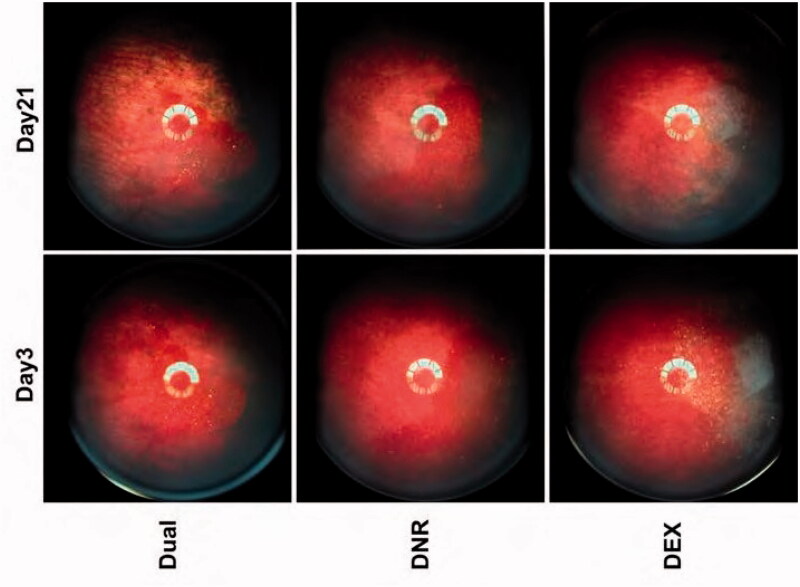
Color fundus images of rabbits at day 3 and day 21, showing the decrease of visible particles over time.

The total mass of each of the three formulations injected into the vitreous of the animals was 2.02 ± 0.27 mg, 1.42 ± 0.45 mg, and 2.04 ± 0.48 mg, for pSiO_2_−DNR/DEX, pSiO_2_−DNR and pSiO_2_−DEX, respectively. The distribution of particles was assessed by fundus imaging (Figure 4). DNR-loaded particles had a red appearance due to the intrinsic color of daunorubicin (Figure 4, middle column) and DEX-loaded particles appeared white to light yellow (Figure 4, right column) in the vitreous; the dual-loaded particles had a less pronounced reddish color compared with the DNR-loaded particles (Figure 4, left column). The particles slowly diminished over time in the vitreous (Figure 4). No vitreous or retina abnormality was noted.

**Figure 5.**
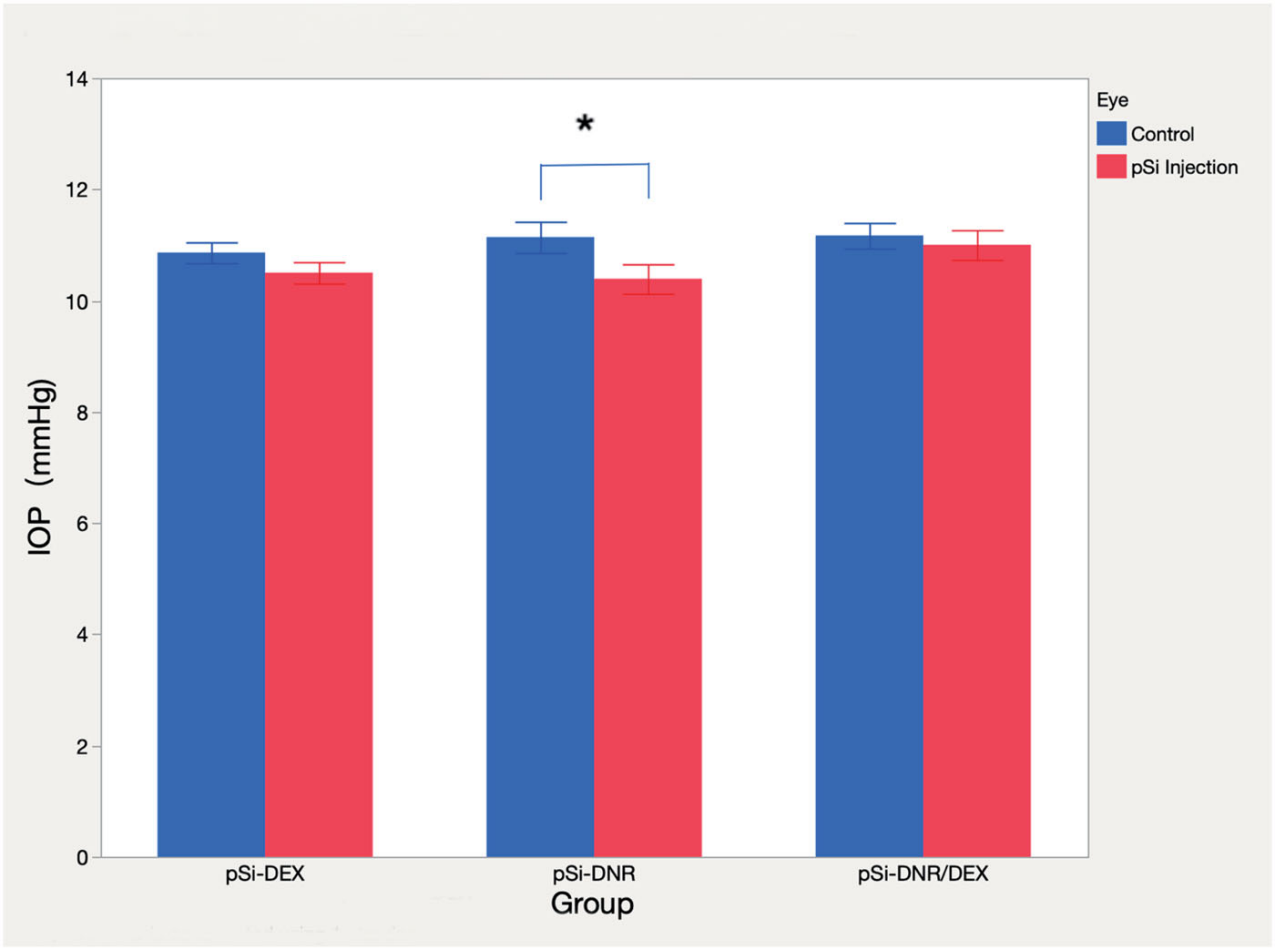
IOP recorded from multiple times were pooled for statistics. Mean IOP of the injected eyes versus the contralateral eyes is being presented above, stratified by groups. The sign ‘*’ indicates statistical significance.

**Figure 6.**
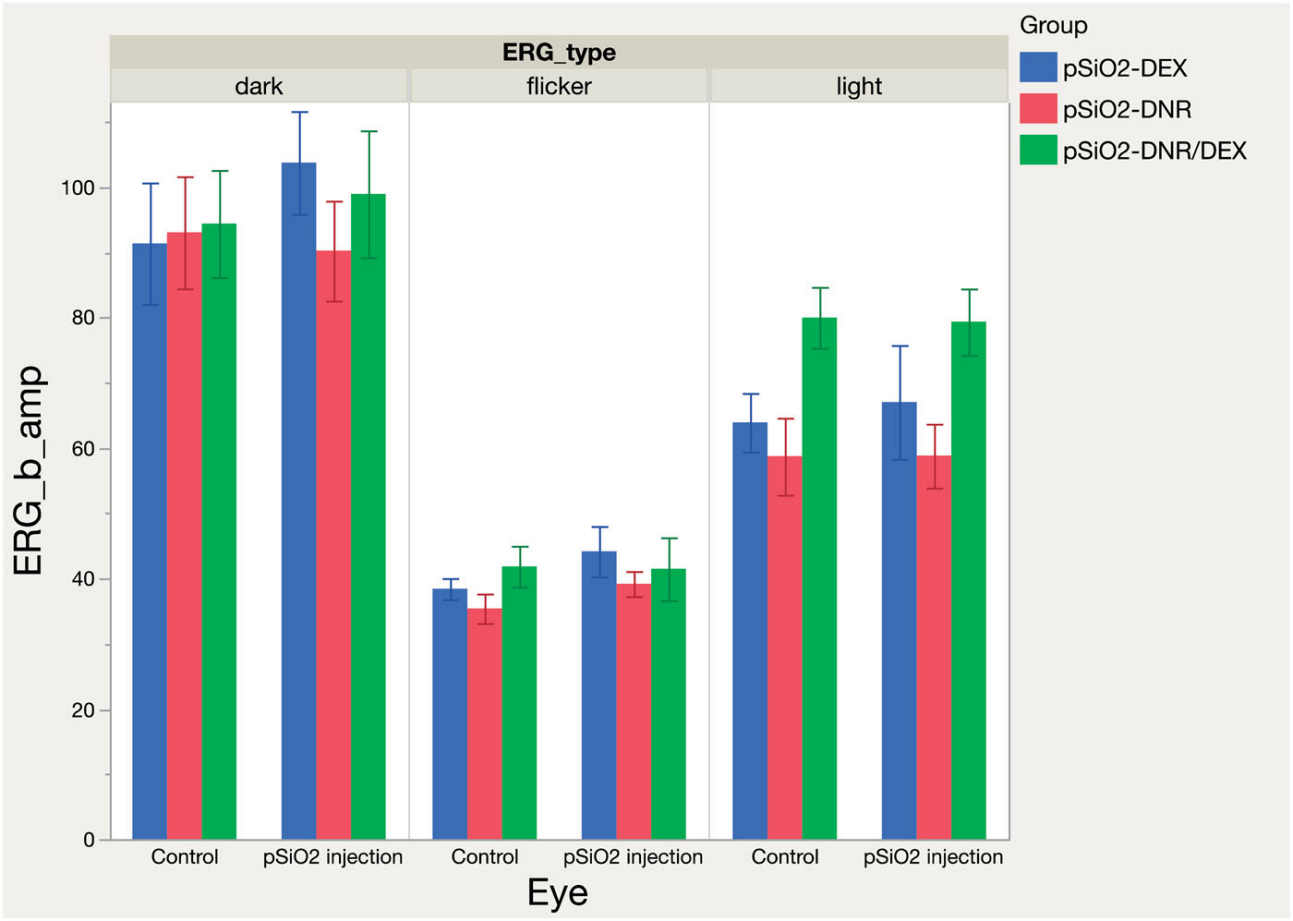
Mean b-wave amplitude values, in microvolts, obtained by electroretinograms (ERGs) measurements in pSiO_2_ injected eyes and control eyes for different groups. ERGs were performed at day 21 after the intravitreal injection prior to creation of the localized retinal detachment.

**Figure 7.**
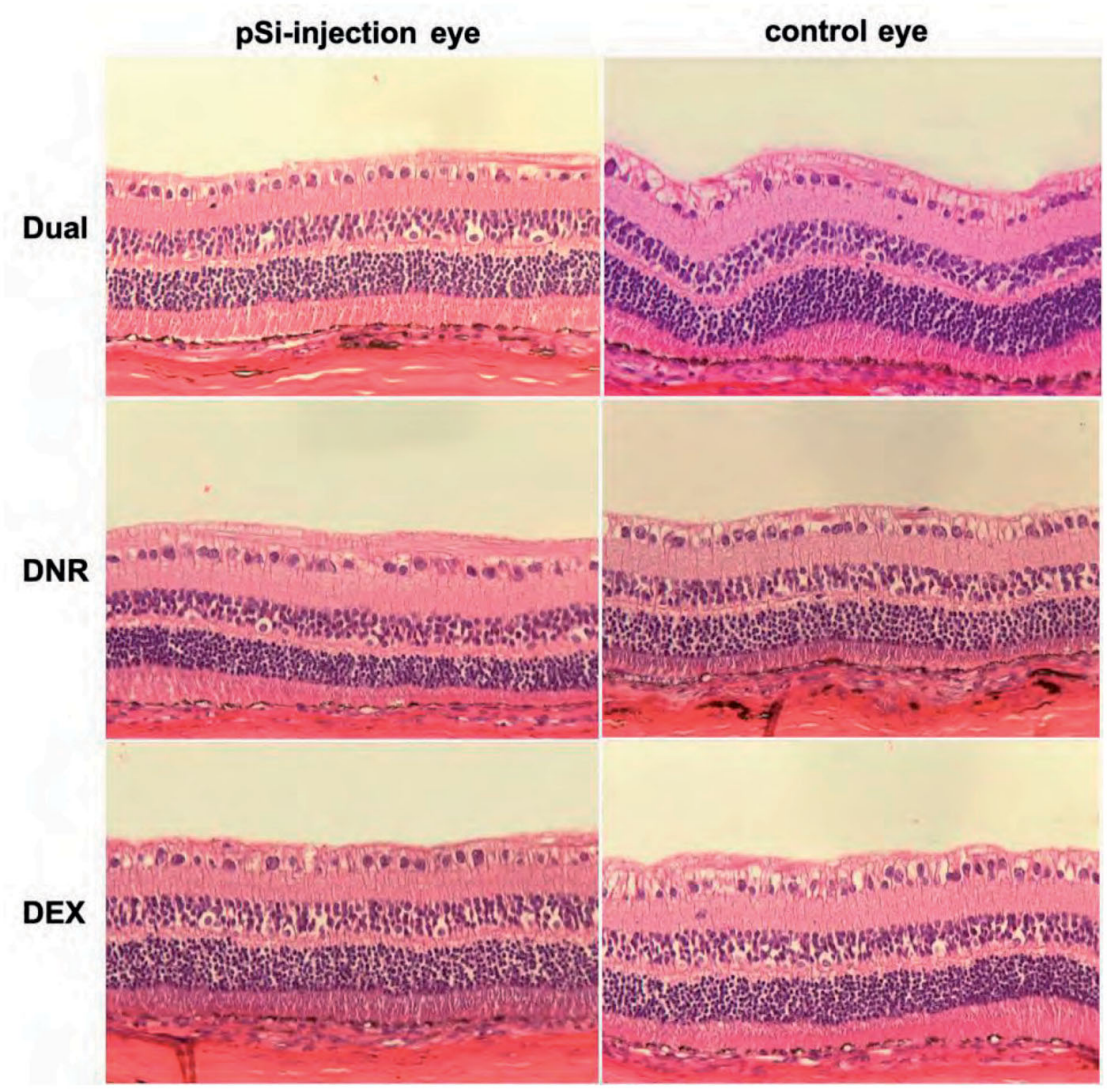
Light microscope images showing normal retina and choroid for each group (20×, H&E staining).

The average IOP value of drug-injected eyes was similar to the BSS-injected control eyes for the Dual and DEX groups (Dual group 11 ± 1.6 mmHg vs. 11.17 ± 1.38 mmHg, and DEX group 10.5 ± 1.16 mmHg vs. 10.86 ± 1.13 mmHg. *p* > .05). IOP of eyes in DNR group was lower than that of their fellow eyes (10.39 ± 1.59 mmHg vs. 11.14 ± 1.68 mmHg, *p* = .0005) with statistical significance (Figure 5). Electroretinograms (ERGs) were performed before the retinal detachment procedure (Figure 6). The b-wave amplitude of dark-adapted, light-adapted, and flicker ERGs revealed no difference between drug injected eyes and control eyes for all 3 groups (*p* > .05). Histology of non-detached retina near the visual streak was compared with the equivalent location from the contralateral eye (Figure 7) and no differences were noted between the two groups.

#### Three-day clinical course of experimental RD in the model eyes

**Figure 8.**
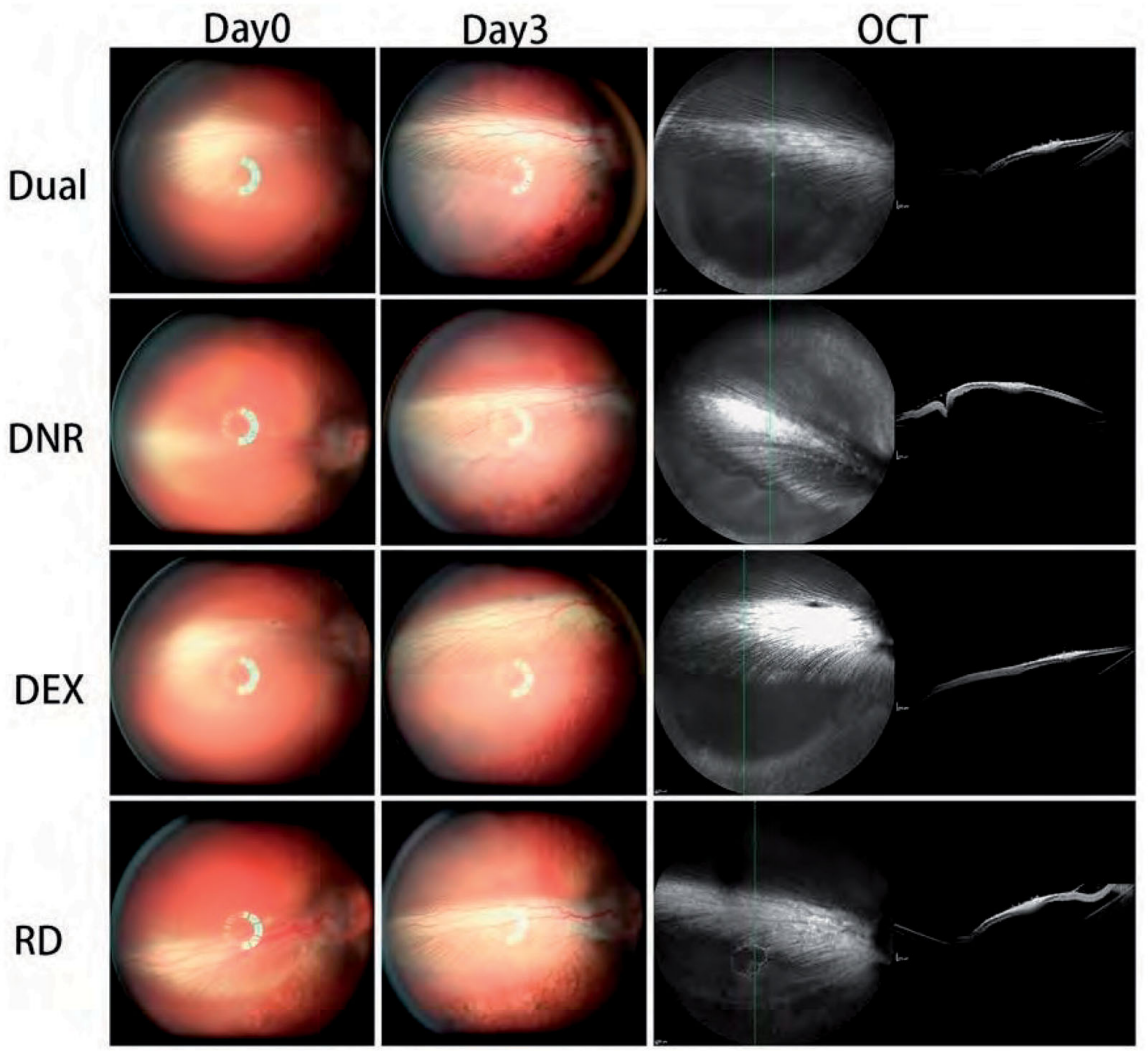
Fundus photograph taken immediately after retinal detachment induction and 3 days later. OCT photos were taken 3 days after retinal detachment. It indicated that retinal detachments were comparable and reproducible cross groups.

Experimentally induced retinal detachment was successful in the nasal retina in all eyes of all groups including control eyes (Figure 8, Day 0 column). The round detached area was located nasal to the optic nerve head, straddling the nasal side of the medullary ray, and measured 5–6 optic disk sizes in diameter. The detachment was still recognizable on day 3 (Figure 8, Day 3 column) and was confirmed by OCT imaging (Figure 8, OCT column).

### Immunohistology of the retina in eyes with different pretreatments

GFAP staining represents the activation of retinal glial cells, which is the hallmark of early cell activity in PVR development. GFAP staining was largely negative in the control eyes except for in the myelinated axons of the medullary ray.

**Figure 9.**
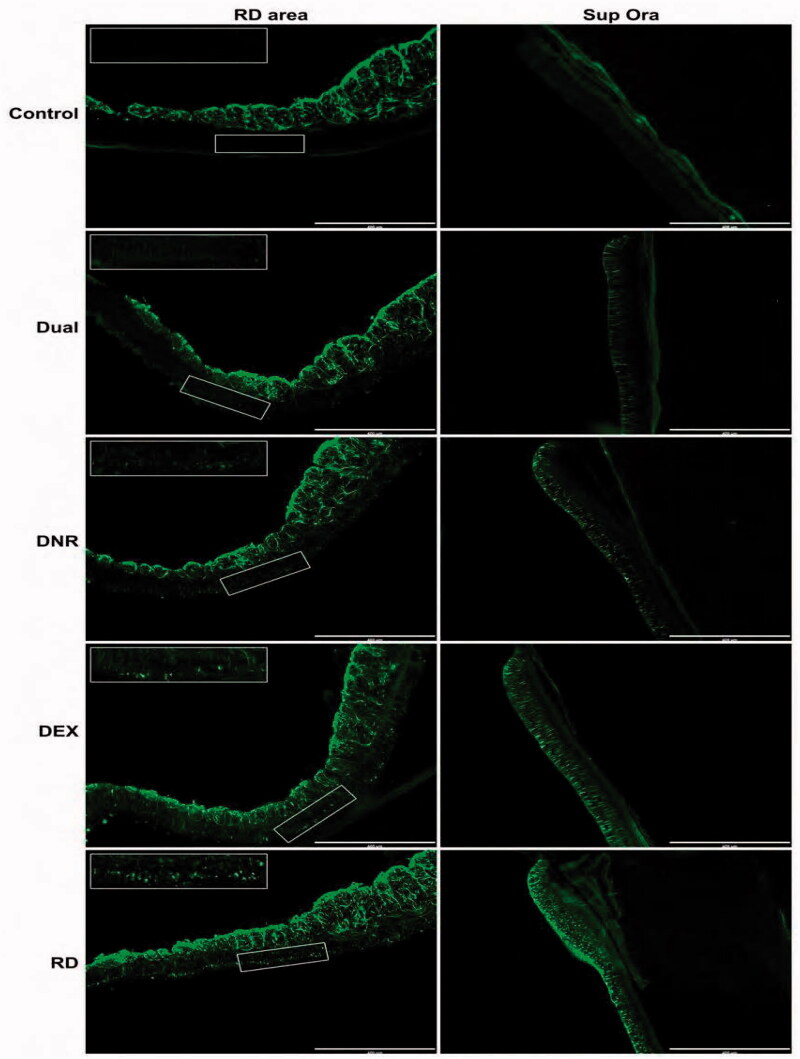
GFAP immune-staining in normal control eye and retinal detachment eyes in each group 3 days after retinal detachment. Left column: detached retina; right column: non-detached retina from the superior ora serrata. (10×) The measured GFAP staining was normalized by the equivalent location of the contralateral eye of the same rabbit.

Therefore, GFAP staining was quantified from the area beneath the myelinated axon layer as shown in the left column of Figure 9. For this study, GFAP staining was quantitated from the whole section of the retina, including detached area and non-detached superior and inferior retina (Table 2).

**Table 2.** GFAP least square mean intensity at different retinal locations (pixel mm^−2^).

	Area		
Group	RD	Superior Non-RD	Inferior Non-RD
Dual	4.03	11.47	6.09
DNR	7.76	18.38	6.09
DEX	16.23	28.94	16.93
RD Control	29.11	49.01	28.05

**Table 3.** Mean number of BrdU-positive cells from RD and non-RD retina (cell mm^−1^).

	Within RD area	Connecting letters report*	Non-RD area	Connecting letters report*
pSiO_2_−DNR + DEX	2.77 ± 1.55	A	0.09 ± 0.11	A
pSiO_2_−DNR	4.58 ± 1.82	B	0.48 ± 0.27	B
pSiO_2_−DEX	4.01 ± 1.65	B C	0.23 ± 0.13	A
RD Control	6.16 ± 2.13	C	0.98 ± 0.55	C

*Levels not connected by same letter are significantly different, Student’s *t*-test, *α* = .05.

Table 2 summarizes the mean intensity of normalized GFAP staining at different locations of the retina. Log transformed GFAP intensity was used as a dependent variable and regression on group and location in a mixed model. The analysis revealed that GFAP intensity was decreasing in the order of Dual drug group, DNR group, DEX group, and Control group (statistically significant from each other, LSMeans differences student’s *t*-test). Location wise, the superior retina had significantly higher GFAP staining intensity than that in the detached retina and the inferior non-detached retina (LSMeans differences student’s *t*-test). The last two locations were not significantly different.

**Figure 10.**
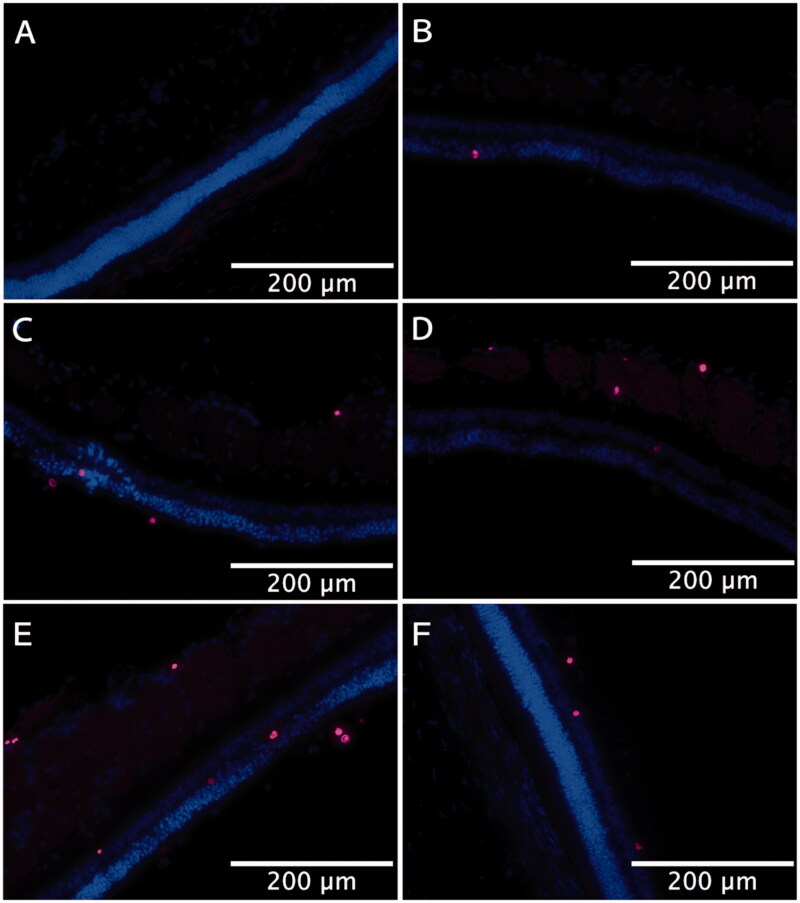
Representative photos of pink BrdU-positive cells from detached area and non-detached area 3 days after retinal detachment from each group (20×). (A) Medullary ray area in normal control retina. (B) Medullary ray area from detached retina of Dual group (pSiO_2_−DNR + DEX). (C) medullary ray area from detached retina of pSiO_2_−DNR group. (D) Medullary ray area from detached retina of pSiO_2_−DEX group. (E) Medullary ray area from detached retina of RD group (control). (F) Peripheral non-detached retina of RD group.

To measure cell proliferation, BrdU-labeled cells (pink) were counted after immunohistochemical staining from both detached retina and non-detached retina. The cells having specific immunostaining were all counted no matter the cells reside in which layer of the retina (Figure 10) and are presented in Table 3. The number of BrdU labeled cells found in the normal retina was 0.05 ± 0.12 cell mm^−1^. The highest number of BrdU labeled cells were detected in the RD control group. DNR and DEX had similar numbers of BrdU positive cells (*p* > .05) but significantly less than the RD control group (*p* < .05). The Dual group had the lowest number of BrdU labeled cells (*p* < .05).

## Discussion

PVR formation is a complex biological process including inflammation, proliferation, and scarring stages (Kwon et al., 2016). Inflammation starts almost immediately after retinal cells are insulted. In response to the inflammation, certain proteins such as glial fibrillary acidic protein (GFAP) will be upregulated for subsequent development of gliosis and contraction, which are the hallmarks of PVR (Fisher & Lewis, 2003; Fisher et al., 2005). Therefore, pharmacological intervention in the early stages of PVR formation is likely to improve outcomes. Another key consideration around PVR suppression is to target both inflammation and proliferation because the single-faceted approach has not shown significant efficacy in clinical trials with either dexamethasone (Banerjee et al., 2017) or 5-fluoruradine (Charteris et al., 2004; Wickham et al., 2007). In the current study, we formulated a combination therapy using oxidized porous silicon (pSiO_2_) loaded with DEX and DNR simultaneously. We first tested the formulations on an in vitro RPE cell-mediated gel contraction model of PVR. Collagen gel contraction is a classical cell culture model that mimics the PVR process *in vitro*. The model combines cell proliferation and contraction. We optimized the experimental system by lowering the cell concentration to 5 × 10^4^ cells mL^−1^ to have a slower proliferation that is more comparable to what is seen in patients. Additionally, a lower therapeutic drug concentration was used, along with replacing the drug-contained media every day for a longer period of exposure to mimic sustained drug delivery. From our previous study (Hou et al., 2015), pSiO_2_−DNR can provide a 3-month sustained release at 10 ng mL^−1^. In the current study, we tested 10 ng mL^−1^ and a half-log higher concentration of 30 ng mL^−1^. For DEX, EC90 is around 40 ng mL^−1^ for inhibiting proinflammatory gene expression (Zhao et al., 2011). The current study tested both 40 ng mL^−1^ and a higher concentration, 80 ng mL^−1^.

In the current gel-contraction study, both 10 and 30 ng mL^−1^ of DNR demonstrated significant suppression in the assay, while 40 and 80 ng mL^−1^ of DEX did not show significant pharmacological suppression of gel contraction compared with the control. For the 30 and 40 ng mL^−1^ two-drug combinations (DNR + DEX), suppression of gel contraction was equivalent to 30 ng mL^−1^ of DNR. All the other combination formulations showed weaker suppression than either 30 ng mL^−1^ of DNR or 30 ng mL^−1^ DNR + 40 ng mL^−1^ DEX, but significantly better than the control. These findings indicate that DEX alone does not suppress gel contraction, consistent with the general understanding that the pharmacologic effect of DEX is mainly anti-inflammatory in nature; thus the *in vitro* gel contraction model is more useful in probing proliferation rather than inflammation. The main conclusion from this set of experiments is that DEX does not substantially interfere with the ability of DNR to suppress proliferation. As inflammation is a well-known component of human PVR and multiple inflammatory cytokines have been confirmed in aqueous and vitreous humors of human eyes exhibiting PVR (Tosi et al., 2014), the results point to the possibility that co-delivery of DEX with DNR might be beneficial.

To test the hypothesis that DEX and DNR co-delivery might show benefits in PVR, we adopted an *in vivo* retinal detachment-induced PVR rabbit eye model to test the dual-drug loaded delivery platform. Prior studies have shown that activated retinal glial cells can be found within the first day of retinal detachment (Lewis et al., 2010; Kuo et al., 2012) and active cell proliferation is observed 3 days after retinal detachment (Fisher et al., 1991). Glial cell response, especially activation of Müller cells, is widely recognized as gliosis in PVR formation. The precursor of gliosis is upregulated expression of intermediate filament proteins such as glial fibrillary acidic protein (GFAP). Therefore, GFAP was quantitated in this study. In addition, cell proliferation is another hallmark of PVR formation and was quantitated by BrdU uptake and labeling. The current study demonstrated that GFAP expression and BrdU uptake were quiescent in the RD-negative control retina while dramatic upregulation of GFAP and cell proliferation (BrdU labeled cells) were found in the RD-positive control retinas.

Intervention by the dual drug treatment clearly demonstrated the strongest therapeutic benefit of suppressing both GFAP expression and cell proliferation in the detached retina when compared with either single drug treatment or control. As mentioned above, this was not the case in the *in vitro* gel contraction study, where 30 ng mL^−1^ DNR showed similar suppression of gel contraction as the 30 ng mL^−1^ DNR + 40 ng mL^−1^ DEX experiment.

Both DNR and steroids are regarded as promising medications for PVR prevention. Once PVR forms, no medication can reverse the pathology. The pharmacological intervention must be applied early, such as during surgical removal of primary PVR or immediately after eye globe trauma, to prevent subsequent PVR development. The current study design was configured as a pretreatment, in which the intervention was applied prior to the induction of the disease model. This is relevant to patients who experience recurrence of PVR after vitrectomy (Mietz & Heimann, 1995). DNR has been used in clinical trials for PVR prevention; however, the application was within a limited time window (10 min) at a low concentration (7.5 µg mL^−1^) due to its cytotoxicity. The result of that clinical trial demonstrated only a very mild preventative effect (Wiedemann et al., 1998). We hypothesized that a sustained delivery system would maximize the pharmacologic effect of DNR at a much lower dose due to the constant presence of DNR in the targeted tissue as shown in our previous publication (Hou et al., 2015). In the current study, we used a dual drug-loaded, oxidized porous silicon system which had a lower total quantity of administered DNR (10 ng mL^−1^) (Warther et al., 2018) relative to the previous DNR-only release system (between 200 and 20 ng mL^−1^) (Hou et al., 2015). The consideration was that the combination of DNR and DEX may reduce the therapeutic concentration of DNR needed, increasing the margin of ocular safety. Indeed, the current *in vivo* study confirmed the synergy of these two drugs, showing significantly improved inhibition of GFAP expression and uptake of BrdU by proliferating cells compared to similar or somewhat larger quantities of either DNR or DEX alone. The dual drug delivery system was well tolerated while the eyes with the DNR-only release system showed a very mild lower IOP (0.75 mmHg lower) compared to their fellow eyes.

In the current study, expression of GFAP was detected beyond the detached retina, most notably in the superior peripheral retina. This may be due to the sclerotomy procedure, which was performed at the superior peripheral location. Yoshida et al. reported a similar finding, where GFAP was first noted in the periphery of the retina after lensectomy–vitrectomy surgery in rabbit eyes (Yoshida et al., 1993). These investigators concluded that GFAP was upregulated in response to the surgical insult and also in response to vitreous tamponade. However, this potential complication is less relevant to the present study because GFAP intensity was compared across three treatment groups within correspondingly consistent areas. Even for GFAP found in the superior peripheral retina, the therapeutic effect was consistent: the dual-drug-pretreated eyes showed the lowest levels of GFAP expression.

In summary, the porous silicon dioxide-based controlled release system delivering both daunorubicin and dexamethasone evaluated in this work was well tolerated in rabbit eyes after intravitreal injection, and it displayed significantly better performance in a rabbit RD model in terms of cellular proliferation and markers of gliosis associated with PVR formation relative to a vehicle delivering or daunorubicin and dexamethasone alone. Our previous study demonstrated that this formulation released both drugs for at least 90 days (Warther et al., 2018). This time window has been considered to be critical for the recurrence of PVR after vitrectomy (Mietz & Heimann, 1995) or for the development of primary PVR after eye globe trauma (Cardillo et al., 1997). Taken together with these previous results, the dual drug delivery system, easy administration by a well-accepted intravitreal injection, and clean degradation of the delivery system (Nieto et al., 2013), may be very useful in the prevention of PVR development.

## References

[CIT0001] Banerjee PJ, Quartilho A, Bunce C, et al. (2017). Slow-release dexamethasone in proliferative vitreoretinopathy: a prospective, randomized controlled clinical trial. Ophthalmology 124:757–67. 28237428 10.1016/j.ophtha.2017.01.021

[CIT0002] Cardillo JA, Farah ME, Mitre J, et al. (2004). An intravitreal biodegradable sustained release naproxen and 5-fluorouracil system for the treatment of experimental post-traumatic proliferative vitreoretinopathy. Br J Ophthalmol 88:1201–5. 15317716 10.1136/bjo.2003.039917 PMC1772295

[CIT0003] Cardillo JA, Stout JT, LaBree L, et al. (1997). Post-traumatic proliferative vitreoretinopathy. The epidemiologic profile, onset, risk factors, and visual outcome. Ophthalmology 104:1166–73. 9224471 10.1016/s0161-6420(97)30167-5

[CIT0004] Case JL, Peyman GA, Barrada A, et al. (1985). Clearance of intravitreal 3H-fluorouracil. Ophthalmic Surg 16:378–81. 4022559

[CIT0005] Charteris DG, Aylward GW, Wong D, et al. (2004). A randomized controlled trial of combined 5-fluorouracil and low-molecular-weight heparin in management of established proliferative vitreoretinopathy. Ophthalmology 111:2240–5. 15582080 10.1016/j.ophtha.2004.05.036

[CIT0006] Cheng L, Hostetler K, Valiaeva N, et al. (2010). Intravitreal crystalline drug delivery for intraocular proliferation diseases. Invest Ophthalmol Vis Sci 51:474–81. 19696179 10.1167/iovs.09-3672 PMC2869063

[CIT0007] Chhablani J, Nieto A, Hou H, et al. (2013). Oxidized porous silicon particles covalently grafted with daunorubicin as a sustained intraocular drug delivery system. Invest Ophthalmol Vis Sci 54:1268–79. 23322571 10.1167/iovs.12-11172 PMC3576052

[CIT0008] Chiba C. (2014). The retinal pigment epithelium: an important player of retinal disorders and regeneration. Exp Eye Res 123:107–14. 23880527 10.1016/j.exer.2013.07.009

[CIT0009] Fisher SK, Erickson PA, Lewis GP, Anderson DH. (1991). Intraretinal proliferation induced by retinal-detachment. Invest Ophth Vis Sci 32:1739–48. 2032796

[CIT0010] Fisher SK, Lewis GP, Linberg KA, Verardo MR. (2005). Cellular remodeling in mammalian retina: results from studies of experimental retinal detachment. Prog Retin Eye Res 24:395–431. 15708835 10.1016/j.preteyeres.2004.10.004

[CIT0011] Fisher SK, Lewis GP. (2003). Müller cell and neuronal remodeling in retinal detachment and reattachment and their potential consequences for visual recovery: a review and reconsideration of recent data. Vision Res 43:887–97. 12668058 10.1016/s0042-6989(02)00680-6

[CIT0012] Hou H, Huffman K, Rios S, et al. (2015). A novel approach of daunorubicin application on formation of proliferative retinopathy using a porous silicon controlled delivery system: pharmacodynamics. Invest Ophthalmol Vis Sci 56:2755–63. 25829415 10.1167/iovs.15-16526 PMC4416660

[CIT0013] Hou H, Wang C, Nan K, et al. (2016). Controlled release of dexamethasone from an intravitreal delivery system using porous silicon dioxide. Invest Ophthalmol Vis Sci 57:557–66. 26882530 10.1167/iovs.15-18559 PMC4758302

[CIT0014] Kaneko H, Shimizu H, Tsunekawa T, et al. (2018). The relationship between inflammatory cytokines in the sub-silicone oil fluid and retinal thickness in eyes with proliferative vitreoretinopathy and proliferative diabetic retinopathy. Invest Ophth Vis Sci 59:5307.

[CIT0015] Kizhakkethara I, Li X, el-Sayed S, et al. (1995). Noninvasive monitoring of intraocular pharmacokinetics of daunorubicin using fluorophotometry. Int Ophthalmol 19:363–7. 8970871 10.1007/BF00130856

[CIT0016] Kuo HK, Chen YH, Wu PC, et al. (2012). Attenuated glial reaction in experimental proliferative vitreoretinopathy treated with liposomal doxorubicin. Invest Ophthalmol Vis Sci 53:3167–74. 22491400 10.1167/iovs.11-7972

[CIT0017] Kwon OW, Song JH, Roh MI. (2016). Retinal detachment and proliferative vitreoretinopathy. Dev Ophthalmol 55:154–62. 26501375 10.1159/000438972

[CIT0018] Lewis GP, Chapin EA, Byun J, et al. (2009). Muller cell reactivity and photoreceptor cell death are reduced after experimental retinal detachment using an inhibitor of the Akt/mTOR pathway. Invest Ophthalmol Vis Sci 50:4429–35. 19369237 10.1167/iovs.09-3445

[CIT0019] Lewis GP, Chapin EA, Luna G, et al. (2010). The fate of Muller’s glia following experimental retinal detachment: nuclear migration, cell division, and subretinal glial scar formation. Mol Vis 16:1361–72. 20664798 PMC2905639

[CIT0020] Mietz H, Heimann K. (1995). Onset and recurrence of proliferative vitreoretinopathy in various vitreoretinal diseases. Brit J Ophthalmol 79:874–7. 7488572 10.1136/bjo.79.10.874 PMC505285

[CIT0021] Nieto A, Hou H, Sailor MJ, et al. (2013). Ocular silicon distribution and clearance following intravitreal injection of porous silicon microparticles. Exp Eye Res 116:161–8. 24036388 10.1016/j.exer.2013.09.001 PMC3873878

[CIT0022] Pennock S, Haddock LJ, Eliott D, et al. (2014). Is neutralizing vitreal growth factors a viable strategy to prevent proliferative vitreoretinopathy? Prog Retin Eye Res 40:16–34. 24412519 10.1016/j.preteyeres.2013.12.006

[CIT0023] Tosi GM, Marigliani D, Romeo N, Toti P. (2014). Disease pathways in proliferative vitreoretinopathy: an ongoing challenge. J Cell Physiol 229:1577–83. 24604697 10.1002/jcp.24606

[CIT0024] Wang C, Hou H, Nan K, et al. (2014). Intravitreal controlled release of dexamethasone from engineered microparticles of porous silicon dioxide. Exp Eye Res 129:74–82. 25446320 10.1016/j.exer.2014.11.002 PMC4259850

[CIT0025] Warther D, Xiao Y, Li FT, et al. (2018). Porous silicon based intravitreal platform for dual-drug loading and controlled release towards synergistic therapy. Drug Deliv 25:1537–45. 29996687 10.1080/10717544.2018.1486474 PMC6058705

[CIT0026] Wickham L, Bunce C, Wong D, et al. (2007). Randomized controlled trial of combined 5-fluorouracil and low-molecular-weight heparin in the management of unselected rhegmatogenous retinal detachments undergoing primary vitrectomy. Ophthalmology 114:698–704. 17398320 10.1016/j.ophtha.2006.08.042

[CIT0027] Wiedemann P, Hilgers RD, Bauer P, Heimann K. (1998). Adjunctive daunorubicin in the treatment of proliferative vitreoretinopathy: results of a multicenter clinical trial. Daunomycin Study Group. Am J Ophthalmol 126:550–9. 9780100 10.1016/s0002-9394(98)00115-9

[CIT0028] Willis JR, Doan QV, Gleeson M, et al. (2017). Vision-related functional burden of diabetic retinopathy across severity levels in the United States. Jama Ophthalmol 135:926–32. 28750122 10.1001/jamaophthalmol.2017.2553 PMC5710538

[CIT0029] Wubben TJ, Besirli CG, Zacks DN. (2016). Pharmacotherapies for retinal detachment. Ophthalmology 123:1553–62. 27040150 10.1016/j.ophtha.2016.02.040

[CIT0030] Yoshida A, Ishiguro S, Tamai M. (1993). Expression of glial fibrillary acidic protein in rabbit Muller cells after lensectomy-vitrectomy. Invest Ophth Vis Sci 34:3154–60. 8407224

[CIT0031] Yu Z, Ma S, Wu M, et al. (2020). Self-assembling hydrogel loaded with 5-FU PLGA microspheres as a novel vitreous substitute for proliferative vitreoretinopathy. J Biomed Mater Res Part A.doi: 10.1002/jbm.a.36995. 32419359

[CIT0032] Zahn G, Volk K, Lewis GP, et al. (2010). Assessment of the integrin alpha5beta1 antagonist JSM6427 in proliferative vitreoretinopathy using in vitro assays and a rabbit model of retinal detachment. Invest Ophthalmol Vis Sci 51:1028–35. 19815730 10.1167/iovs.09-3575

[CIT0033] Zhao M, Bousquet E, Valamanesh F, et al. (2011). Differential regulations of AQP4 and Kir4.1 by triamcinolone acetonide and dexamethasone in the healthy and inflamed retina. Invest Ophth Vis Sci 52:6340–7. 10.1167/iovs.11-7675 21724913

